# John Gamgee

**Published:** 1895-04

**Authors:** 


### OBITUARY.

### JOHN GAMGEE.

John Gamgee, who died on December 19, 1894, was a son of the eminent veterinarian, Mr. Joseph Gamgee, who still survives, and has now reached the age of ninety-three, but retains the full vigor of his health and great intellect.

John Gamgee was born in Florence, Italy, May 3, 1831. His father at an early age obtained an appointment in Italy, where he spent a number of years in gaining knowledge and experience from the celebrated schools there. His son John was sent to study anatomy under Professor Pacini, at Florence, and was intended for the medical profession, but changed his course of studies to that of veterinary medicine. He later went to the Imperial School at Alfort, and to the Royal Veterinary College in London, graduating from the latter May 26, 1852. In 1856 he went to Edinburgh as an assistant to Professor Dick, and there filled the Chair of Anatomy with great credit and with great popularity amongst the students. He was devoted to his work, assiduous in his duties, and lectured twice almost daily, on both anatomy and physiology. Mr. Gamgee at an early period of his life manifested a decided aptitude for engineering, which interested him throughout his whole career, and became the object of all his energy when he severed his connection with the veterinary profession. Mr. Gamgee was a man of great refinement and the most polished manners. He was tall, well built, and of fine appearance, and was greatly esteemed by a wide circle of friends.

